# Nutrition Support for Head and Neck Squamous Cell Carcinoma Patients Treated with Chemoradiotherapy: How Often and How Long?

**DOI:** 10.5402/2012/274739

**Published:** 2012-02-13

**Authors:** Hiroto Ishiki, Yusuke Onozawa, Takashi Kojima, Shuichi Hironaka, Akira Fukutomi, Hirofumi Yasui, Kentaro Yamazaki, Keisei Taku, Nozomu Machida, Narikazu Boku, Takayuki Hashimoto, Tetsuo Nishimura

**Affiliations:** ^1^Division of Gastrointestinal Oncology, Shizuoka Cancer Center, 1007 Shimonagakubo, Nagaizumi-cho, Sunto-gun Shizuoka 411-8777, Japan; ^2^Division of Radiation Oncology, Shizuoka Cancer Center, 1007 Shimonagakubo, Nagaizumi-cho, Sunto-gun Shizuoka 411-8777, Japan

## Abstract

*Background.* Oral intake of many patients with locally advanced head and neck cancer (LAHNC) decrease during chemoradiotherapy (CRT). Although prophylactic percutaneous endoscopic gastrostomy (PEG) is recommended, not a few patients complete CRT without using PEG tube. *Patients and Methods.* The subjects were patients with LAHNC who received CRT. We retrospectively investigated the incidence and duration of nutritional support during and after CRT, and predicting factors of nutritional support. For patients who required nutritional support, we also checked the day of initiation and the duration of nutritional support. *Results.* Of 53 patients, 29 patients (55%) required nutritional support during and/or after CRT. While no clear relation between requirement of nutritional support and variables including age, T stage, N stage, clinical stage and chemotherapy regimen, there could be some relationships between tumor primary sites and the requirement and duration of nutritional support. 17 (77%) of 22 patients with oropharynx cancer(OP) required nutritional support and prolonged for 4.4 months, and 11 (46%) of 24 patients with hypopharynx cancer(HP) required nutritional support and prolonged for 21.9 months. *Conclusion.* Nutritional support is indicated many HNC patients treated with CRT and primary sites may have some relation to its indication and duration.

## 1. Introduction

Chemoradiotherapy (CRT) is one of the treatment choices for locally advanced head and neck squamous cell carcinoma (HNCSCC), not only for patients with unresectable disease, but also for those who desire organ preservation. However, the treatment course is often complicated by the development of painful mucositis, which causes difficulty in oral intake. Furthermore, in some patients, dysphagia occurring as a result of CRT causes life-threatening aspiration pneumonia during and/or after treatment [1, 2]. These obstacles to oral intake often result in treatment failure, prolongation of hospitalization, and treatment-related death [3]. Many physicians have begun to pay more attention to these adverse effects and to developing means to overcome these adverse effects and support the patients' nutrition during and after CRT [4]. While placement of a percutaneous endoscopic gastrostomy (PEG) or gastric feeding tube (GFT) before CRT is recommended in Western countries, prophylactic placement of a GFT has generally not been accepted in Japan. In addition, some patients do not require nutritional support at all. It would be reasonable to carefully select patients in whom PEG or GFT should be considered prior to CRT.

There are only few reports concerning nutritional support for HNSCC patients treated by CRT [5]. Especially, there have been no reports about the relationship between the indications and duration of nutritional support and the clinical backgrounds of HNSCC patients. This is the first report on the incidence and duration of nutritional support during and after CRT in patients with HNSCC and of determination of the indication for nutritional support according to the clinical backgrounds of the patients.

## 2. Subjects and Methods

### 2.1. Subjects

Patients with HNSCC receiving CRT as the first-line therapy at Shizuoka Cancer Center between 2002 and 2006, who fulfilled the following criteria were enrolled as the subjects of this retrospective study; (1) primary site, oropharynx (OP), hypopharynx (HP), or larynx (L); (2) no other malignancy; (3) completed CRT; (4) adequate oral intake before CRT. The clinical stage of the disease was classified according to the UICC TNM classification.

### 2.2. Treatment

 All patients received concomitant CRT. The total radiation dose was 60–70 Gy, administered in 30–35 conventional fractions. The chemotherapy regimens were selected from the following four regimens, in accordance with the physicians' judgment of the patient's medical condition and the availability of informed consent from the patient; (1) 5-fluorouracil (5-FU, 400 mg/m^2^, ci, days 1–5, days 36–40) plus cisplatin (CDDP, 80 mg/m^2^, div days 1 and 36), (2) CDDP alone (100 mg/m^2^, div, days 1, 22, 43), (3) nedaplatin (90 mg/m^2^, div, days 1 and 36) and 5-FU (400 mg/m^2^, ci, days 1–5, days 36–40), and (4) carboplatin alone (AUC = 5, days 1 and 29). Treatment response was assessed according to the RECIST.

### 2.3. Evaluation of Nutritional Support

The time to provision of nutritional support was calculated from the date of start of the CRT to the date of start of nutritional support. The duration of nutritional support was calculated as the total number of days for which the patients received enteral nutrition (EN) via a feeding tube (nasogastric feeding tube; NGFT or GFT) or total parental nutrition (TPN), because of the lack of ability for adequate oral intake, regardless of the amount of oral intake and calories provided through nutritional support; the days on which the patients received no calories through NGFT/GFT/TPN were not included in the calculation of the duration of nutritional support, regardless of the presence of a PEG, GFT, or central venous catheter in place. The nutritional support-free survival was calculated by subtracting the duration of nutritional support from the overall survival, and the patients were censored when they received surgery for residual tumor or local relapse or died.

### 2.4. Statistics

The relationship between the clinical background characteristics and indication for nutritional support was analyzed by the chi-square test. Then, the durations of nutritional support-free survival and nutritional support were analyzed by the Kaplan-Meier method and log-rank test. 

## 3. Results

### 3.1. Patient Background

A total of 74 patients with HNSCC received CRT as the first-line therapy at our center during the study period. Fourteen patients having double cancer, six patients with insufficient oral intake, and one patient in whom the CRT was not completed were excluded, and the remaining 53 patients were enrolled as the subjects of this study.

The patient characteristics are shown in Table 1. The median age was 62 years, and there were 49 male and 4 female patients. The primary sites were the OP, HP, and L in 22, 24, and seven patients, respectively. Most had advanced disease; T stage 1/2/3/4 in 2/18/12/21 patients, N stage 0/1/2a/N2b/2c/3 in 6/3/2/9/25/8 patients, and clinical stage II/III//IV in 4/3/46 patients.

### 3.2. Treatment

Thirty patients received CDDP and 5-FU, 17 patients received CDDP alone, three received nedaplatin plus 5-FU, and three patients received carboplatin alone. The best overall responses are shown in Table 2. Complete response (CR) was achieved in 28 patients (53%). Stratified according to the primary site, CR was obtained in 11 patients (50%) with primary cancer of the OP, in 13 patients (54%) with the cancer arising from the HP, and 4 (57%) with a primary tumor of the L. The mean survival times were 623 days for patients with tumors of the OP, 1111 days for patients with tumors arising from the HP, and 1408 days for patients with primary tumor of the L.

### 3.3. Nutritional Support

**Figure 1:**
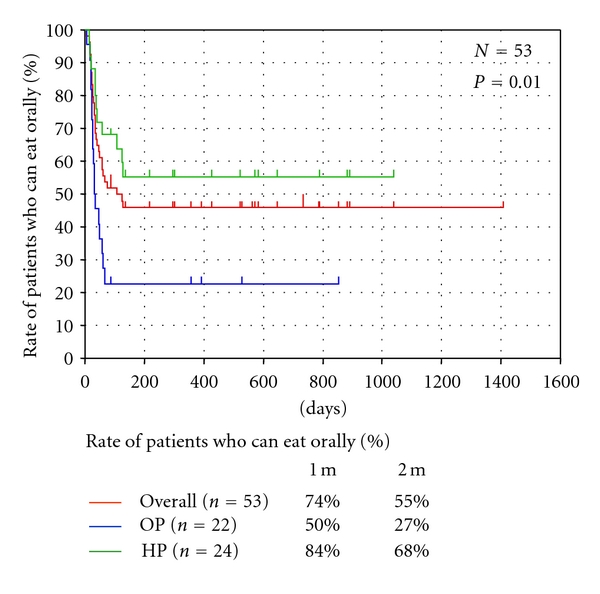


 In this study, the nutritional support-free survival rates at two months from the start of the CRT were 27% for patients with tumors arising from the OP and 64% for patients with tumors arising from the HP. Finally, 77% of the patients with primary tumor of the OP and 46% of the patients with tumor arising from the HP required nutritional support throughout the duration of the CRT (Figure 1).  Table 3 shows the relationship between the clinical background characteristics and the need for nutritional support. In total, 29 patients (55%) required nutritional support. The need for nutritional support was more frequent in patients with tumor arising from the HP than in those with primary tumor of the OP (*P* = 0.02). There were no significant differences in the other variables, including the age, distribution of gender, T stage, N stage, clinical stage or chemotherapy regimens, or the tumor resectability among the groups. A significant difference in the nutritional support-free survival was observed between patients with tumors arising from the OP and HP (*P* = 0.01). The nutritional support-free survival rates at one month were 50% and 84%, and those at two months were 27% and 64%, in the patients with tumors arising from the OP and HP, respectively. Finally, 23% of the patients with primary tumor of the OP and 54% of the patients with primary tumor of the HP did not require nutritional support throughout the duration of the CRT.

**Figure 2:**
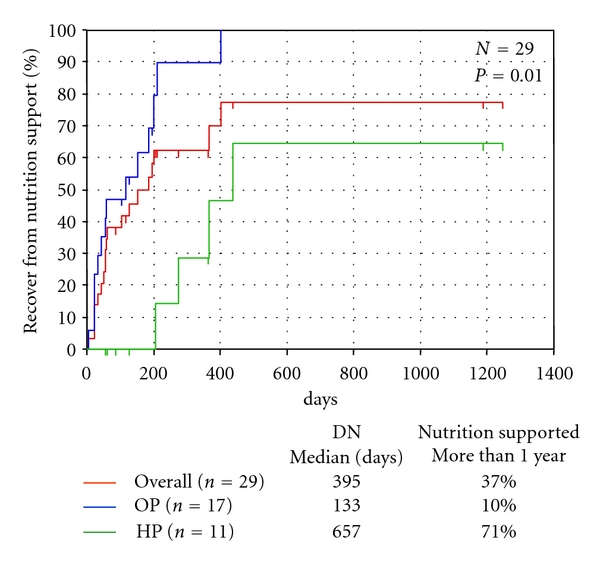


Figure 2 shows the duration of nutritional support according to the primary site of the tumors. The median duration of nutritional support was 133 days in the patients with primary tumor of the OP and 657 days in those with primary tumor of the HP; 10% of patients with primary tumor of the OP and 71% of patients with primary tumor of the HP required nutritional support for more than one year (*P* = 0.002).

## 4. Discussion

In recent years, CRT has come to be recognized as an important treatment modality for locally advanced HNSCC. Especially patients with unresectable disease and those who do not wish to lose their voice by surgery, CRT is the treatment modality with curative intent of first choice. However, CRT is associated with severe toxicities, such as grade 3 or 4 mucositis, and the associated pain and/or burning sensation make oral intake difficult. Placement of a nutritional support device before CRT is generally recommended to improve the feasibility of CRT. Actually, 77% of the patients with primary tumor arising from the OP and 46% of patients with primary tumor arising from the HP required nutritional support throughout the duration of CRT in this study.

Placement of a nutritional support device before CRT is generally recommended to improve the feasibility and likelihood of completion of CRT [6, 7]. However, in quite a few patients, CRT can be completed without provision of any nutritional support. For such patients, placement of a nutritional support device might represent overtreatment. Thus, it is important to identify which patients might actually require nutritional support. In this study, the nutrition support-free survival rates were 27% in the patients with primary tumor of the OP and 64% in those with primary tumor of the HP at two months from the beginning of the CRT; furthermore, 23% of the patients with primary tumor of the OP and 54% of the patients with primary tumor of the HP did not require nutritional support at all. These findings suggest that approximately more than half of the patients with primary tumor arising from the HP did not require nutritional support, while about three quarters of all patients with primary tumor arising from the OP needed nutritional support. Our findings suggest that the site of origin of the primary tumor may be a predictor of the need for nutritional support, while none of the other clinical background characteristics appeared to be related to the need for nutrition support. 

Even after completion of CRT and resolution of the mucositis, some patients suffer from prolonged persistent dysphagia and continue to need nutritional support via tube feeding or TPN, until they recover their ability to swallow ability recovers [8, 9]. In this study, the median duration of nutritional support was 133 days in the patients with primary tumor of the OP and 657 days in patients with primary tumor of the HP. Furthermore, 10% of the patients with primary tumor arising from the OP and 71% of the patients with primary tumor arising from the HP required nutritional support for more than one year. While CRT may be a useful modality for preserving the organ and voice, unfortunately, it may impair the ability for adequate oral intake. Because we did not evaluate the swallowing ability by a barium test before and after the CRT, it is not known precisely why and how the patients complained of difficulty in oral intake. It is speculated that swallowing dysfunction or mental stress may be the reason for the inadequate oral intake, since the organs for swallowing are mechanically preserved [10–12]. The usefulness of additional approaches, such as precise evaluation of the swallowing ability and provision of practice for swallowing throughout the course of the CRT should be investigated to prevent dysphagia.

Furthermore, approximately 30% of patients with primary tumor arising from the HP patients required nutritional support even after the completion of CRT. Once the patients were started on nutritional support, it took 4.4 months for patients with primary tumor arising from the OP and 21.9 months for those with the primary tumor arising from the HP before the patients could eat well enough and the nutritional support could be discontinued. While most patients with primary tumor arising from the oropharynx required nutritional support, the duration of nutritional support was rather short, and only a half of the patients required prolonged nutritional support. It is speculated that mucositis and edema of the oral mucosa, tongue, and pharynx in the patients with primary tumor of the OP during CRT, which make oral intake difficult, resolve soon after CRT without residual dysfunction. On the contrary, patients with primary tumors arising from the HP, damage to the important structures for swallowing, such as the pharyngeal stricture muscles, might be present, or stricture of the pharynx to cervical esophagus might be inflicted by the CRT, increasing the time until recovery of the swallowing functions [13]. Thus, it is suggested that different strategies for nutritional support, taking into consideration the primary tumor site, should be considered.

## 5. Conclusion

Nutritional support for HNSCC patients receiving CRT is very important. The optimal strategy for provision of nutritional support according to the primary site of the tumor should be investigated for preservation of normal swallowing function.

## Figures and Tables

**Table 1:** 

	*n* = 53	
Age	Median (range)	62 (45–77)
Gender	Male/female	49/4
Primary sites	OP/HP/L	22/24/7
T	1/2/3/4	2/18/12/21
N	0/1/2a/2b/2c/3	6/3/2/9/25/8
Stage	II/III/IV	4/3/46
Chemotherapy regimen	CDDP + 5-FU + RT	30
	CDDP + RT	17
	Nedaplatin + 5-FU + RT	3
	Carboplatin + RT	3
RT dose	Median (range)	70 (60–70)

Abbreviations; OP: Oropharynx, HP: Hypopharynx, and L: Larynx.

**Table 2:** 

	CR	non CR	Total
OP	11	11	22
HP	13	11	24
L	4	3	7

Total	28	25	53

Abbreviations; CR: complete response.

**Table 3:** 

	Nutrition supported	Not supported	*P* value
	*n* = 24 (45%)	*n* = 29 (55%)	
Male/female	24/0	25/4	0.21
Age (range)	60 (45–77)	60 (47–74)	0.48*
OP/HP/L	5/13/6	17/11/1	0.02**
T1,2,3/4	16/8	16/13	0.51
N0,1,2a/2b,2c,3	8/16	4/25	0.2
Stage II, III/IV	5/19	2/27	0.15
Chemotherapy regimen (including 5-FU/without 5-FU)	17/13	16/7	0.33
Resectability (yes/no)	10/19	11/13	0.4

*Over 70 versus under 69.

**OP versus HP.
